# A Retrospective Imaging Evaluation of Presynaptic Dopaminergic Degeneration in Multiple System Atrophy with Levodopa Induced Dyskinesia

**DOI:** 10.5334/tohm.58

**Published:** 2020-06-15

**Authors:** Shin-ichi Ueno, Genko Oyama, Kazuaki Kanai, Taku Hatano, Yasushi Shimo, Nobutaka Hattori

**Affiliations:** 1 Department of Neurology, Juntendo University Faculty of Medicine, 2-1-1 Hongo, Bunkyo-ku, Tokyo, JP; 2 Department of Neurology, Fukushima Medical University School of Medicine, 1 Hikarigaoka, Fukushima, Fukushima, JP

**Keywords:** multiple system atrophy, dopamine transporter, burst pattern, levodopa induced dyskinesia

## Abstract

**Background:**

Multiple system atrophy (MSA) may develop levodopa-induced dyskinesia, which is dystonic and predominant in the orofacial region. We aimed to characterize the patterns of presynaptic dopaminergic degeneration in patients with MSA and dyskinesia using ^123^I-N-x-fluoropropyl-2b-carbo-methoxy-3b-(4-iodophenyl) nortropan single-photon emission computed tomography (^123^I-FP-CIT SPECT).

**Methods:**

A single center cross-sectional retrospective study was conducted using consecutive chart review of patients with probable MSA who underwent ^123^I-FP-CIT SPECT. The degeneration patterns were compared between the groups with and without dyskinesia via visual assessment of ^123^I-FP-CIT SPECT images.

**Results:**

Twenty-five patients with probable MSA who had undergone dopamine transporter imaging were identified (age [mean ± standard error], 62.5 ± 1.7 years; disease duration, 48.8 ± 7.0 months). Four of them presented dyskinesia and 21 of patients did not. Twenty-five patients with MSA were visually classified into five grades: one Grade 1 (normal), two Grade 2 (eagle wing), three Grade 3 (mixed), nine Grade 4 (egg shape), and ten Grade 5 (burst striatum). All patients with MSA and dyskinesia were classified into Grade 5. Visual grading significantly correlated with disease duration and levodopa responsiveness.

**Conclusions:**

Severe presynaptic dopaminergic dysfunction in ^123^I-FP-CIT SPECT images, higher doses of dopaminergic medication, and longer disease durations were associated with occurrence of levodopa-induced dyskinesia, even in MSA.

## Introduction

Multiple system atrophy (MSA) is a neurodegenerative disorder characterized by autonomic failure, poor levodopa-responsive parkinsonism, and cerebellar ataxia. It is classified into the Parkinson type (MSA-P), which is parkinsonism predominant, and the cerebellar type (MSA-C), which is cerebellar ataxia predominant [1]. As presynaptic dopaminergic denervation is predominant in Parkinson’s disease (PD), dopamine-replacement therapy is effective. However, not only presynaptic but also postsynaptic dopaminergic denervation starts at a relatively early stage in MSA. Therefore, levodopa is usually less effective, or responsiveness to levodopa weans off earlier, in MSA than in PD [2].

Levodopa-induced dyskinesia (LID) is the major complication of long-term levodopa-replacement therapy for PD. The main pathophysiology of LID has been considered to be the inability for storage and the pulsatile release of dopamine due to the degeneration of striatal presynaptic dopaminergic neurons. Some MSA-P patients treated with levodopa are also known to develop LID [2,3,4]. In general, LID in MSA is typically predominant in axial and craniocervical lesions, and it is dystonic but could be generalized choreic in some cases [3,4]. The detailed mechanisms underlying LID in MSA remain unclear.

Single-photon emission computed tomography (SPECT) of dopamine transporter (DAT) utilizing ^123^I-N-x-fluoropropyl-2b-carbo-methoxy-3b-(4-iodophenyl)nortropan (^123^I-FP-CIT) is useful for detecting nigrostriatal degenerative disorders [5]. Kahraman et al. proposed a visual assessment of ^123^I-FP-CIT SPECT images, and showed different patterns for each parkinsonian syndrome [6]. A recent study showed that presynaptic dopamine depletion visualized using DAT-SPECT predicts LID in PD [7]. In this study, we aimed to characterize the patterns of presynaptic dopaminergic degeneration in patients with MSA and dyskinesia using ^123^I-FP-CIT SPECT.

## Methods

We retrospectively reviewed the records of patients who satisfied Gilman’s criteria for probable MSA and underwent ^123^I-FP-CIT SPECT, which is usually performed as a regular work-up for parkinsonism and other neurodegenerative disorders, between March 2014 and May 2015 [8]. The diagnosis of MSA-P and MSA-C was made on the basis of the clinically predominant phenotype, regardless of imaging findings. All patients with MSA had been treated as outpatients at Juntendo University and confirmed antiparkinsonian drugs for acquisition of ^123^I-FP-CIT SPECT. The data collected from the chart included age, sex, age at onset, disease duration, the Unified Parkinson’s Disease Rating Scale (UPDRS) score in the on-state, Hoehn & Yahr stage, levodopa responsiveness (subjective and/or objective), levodopa dose (LDD), levodopa equivalent dose (LED), body weight, and the average specific binding ratio (SBR) of ^123^I-FP-CIT SPECT. This retrospective study was approved by the institutional review board in Juntendo University Hospital. Movement disorder specialists (GO, KK, and HT) performed clinical assessments including levodopa responsiveness and presence of LID. ^123^I-FP-CIT SPECT was performed using a dual-head gamma camera (E. Cam Signature; Symbia E/Symbia S, Low-Medium Energy General Purpose [LMEGP] collimator, SIEMENS, Munich, Germany), 3 hours after the intravenous injection 167 MBq of ^123^I-FP-CIT (Ioflupane injection; Nihon Medi-Physics Co., Ltd., Tokyo, Japan). The gamma camera was calibrated for a 150-keV photo peak and ±20% energy window. Parameters for the dynamic study were as follows: 4/projection; 2.5 min/cycle × 10 cycles, and continuous mode.

**Figure 1:**
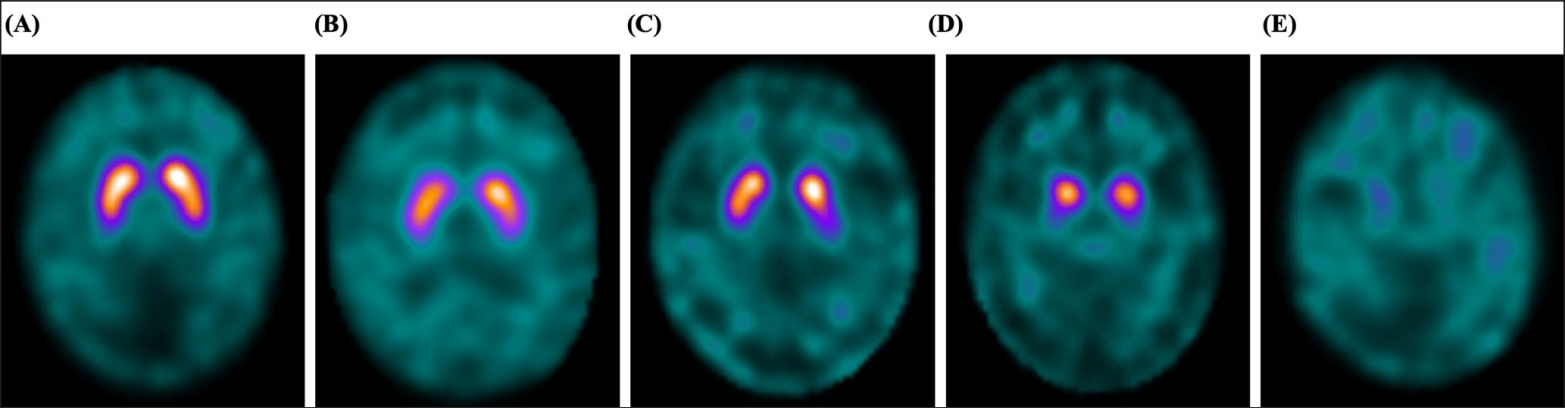
**Kahraman’s visual assessment in ^123^I-FP-CIT SPECT images. (A)** Grade 1 (normal), **(B)** Grade 2 (eagle wing), **(C)** Grade 3 (mixed), **(D)** Grade 4 (egg shape), and **(E)** Grade 5 (burst striatum).

On ^123^I-FP-CIT SPECT images, striatal dopaminergic deficit was estimated using the SBR of the voxel of interest (VOI) on the striatum (DaTView software; Nihon Medi-Physics Co., Ltd., Tokyo). First, the striatum was preconditioned; thereafter, setting of VOI on striatum and reference VOI were conditioned, finally calculated the SBR [5]. We then classified the visual patterns on the ^123^I-FP-CIT SPECT images into five grades utilizing Kahraman’s visual assessment: [6] Grade 1 (normal), Grade 2 (eagle wing), Grade 3 (mixed), Grade 4 (egg shape), and Grade 5 (burst striatum) (Figure 1). The ^123^I-FP-CIT SPECT images were evaluated by experienced movement disorder specialists (GO, KK, and HT). The characteristics of ^123^I-FP-CIT SPECT images were compared between patients with and without LID.

## Statistical analysis

Patient characteristics were analyzed using Student’s t-test, chi-square test and Fisher’s exact test. Correlations between visual assessment grades and each parameter were calculated using Pearson’s correlation coefficient. A two-sided P value < 0.05 was considered significant. We used JMP 13 (SAS Institute) to perform statistical analysis.

## Results

**Figure 2:**
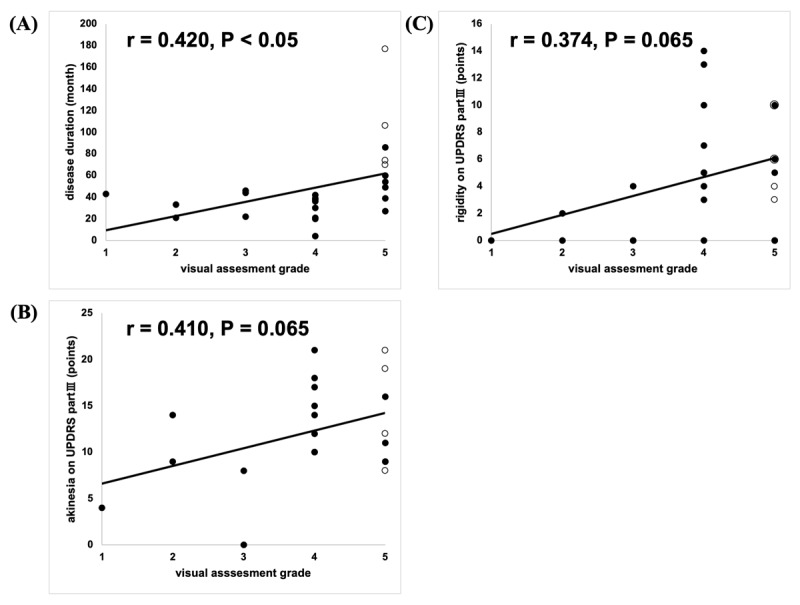
**Correlation of dopamine transporter single-photon emission computed tomography visual assessment grade and disease duration. (A)** disease duration, **(B)** bradykinesia (finger tapping, rapid alternating movement, and foot tapping), **(C)** rigidity (neck and limbs). Open circle; MSA patients with dyskinesia, closed circle; MSA patients without dyskinesia. p Value was obtained by Pearson’s correlation coefficient. Abbreviation: UPDRS: Unified Parkinson’s Disease Rating Scale.

We identified 25 patients with probable MSA who had undergone DAT imaging. The characteristics of these patients are shown in Table 1. Their average age was 59.3 ± 2.13 (standard error [SE]) years, age at onset was 50.3 ± 3.00 (SE) years, and disease duration was 106.8 ± 24.8 (SE) months. Among the 25 patients, four presented LID and 21 did not. Patients with LID had significantly longer disease duration, higher LED, better levodopa responsiveness, and lower SBRs. Based on Kahraman’s visual assessment [6], the 25 patients were visually classified into the five grades as follows: one Grade 1 (normal), two Grade 2 (eagle wing), three Grade 3 (mixed), nine Grade 4 (egg shape), and ten Grade 5 (burst striatum). All patients with MSA and dyskinesia were classified into Grade 5. We also conducted a sub-analysis of patients with burst pattern, and it revealed that patients with LID had, high responsiveness to levodopa, higher LED, lower SBRs, and longer disease duration than patients without LID. Visual assessment grading of DAT-SPECT images significantly correlated with clinical parameters such as disease duration (r = 0.420, P < 0.05). The grading also correlated with disease duration in MSA-P (MSA-P: disease duration; r = 0.686, P = 0.0047), but not in MSA-C (r = 0.438, P = 0.204). The visual assessment grading did not correlate with clinical symptoms such as bradykinesia (r = 0.410, P = 0.065) and rigidity (r = 0.374, P = 0.065; Figure 2), and in MSA clinical subtypes either (MSA-C: bradykinesia; r = 0.537, P = 0.135, rigidity; r = 0.592, P = 0.0925, MSA-P: bradykinesia; r = 0.0639, P = 0.821, rigidity; r = -0.261, P = 0.347).

**Table 1:** Patients characteristics.

	With LID (n = 4)	Without LID (n = 21)	P value

**Age (years)**	59.3 ± 2.14	63 ± 1.89	0.411
**Sex (M/F), n**	0/4	11/10	0.09^a^
**Disease duration (months)**	106.8 ± 24.8	48.8 ± 6.98	<0.001
**Age at onset (months)**	50.3 ± 3	59.8 ± 1.98	0.057
**Duration of LID (months)**	66.7 ± 15.9		
**MSA-C/MSA-P**	1/3	9/12	0.627
**Levodopa responsiveness**	4/4	9/21	<0.05
**Hoehn & Yahr stage**	4.5 ± 0.29	3.7 ± 0.14	0.130
**Rigidity on UPDRS part III**	5.75 ± 1.54	4.48 ± 0.96	0.587
**Akinesia on UPDRS part III**	15.3 ± 2.84	11.5 ± 1.27	0.222
**LDD (mg)**	600 ± 141	426 ± 98	0.449
**LDD/body weight (mg/kg)**	15.8 ± 3.6	7.8 ± 2.0	0.122
**LED (mg)**	1053 ± 158	494.6 ± 495	0.0424
**LED/Body weight (mg/kg)**	25.9 ± 8.7	9.95 ± 11	0.0162
**Specific binding ratio (average)**	0.64 ± 0.42	2.54 ± 0.36	<0.05

**Abbreviations:** LID: levodopa-induced dyskinesia, MSA-C: cerebellar-type multiple system atrophy, MSA-P: Parkinson-type multiple system atrophy, UPDRS: Unified Parkinson’s Disease Rating Scale, LDD: levodopa dose, LED: levodopa equivalent dose. p Value was obtained by t-test. p Value^a^ was obtained by fisher’s exact test (median (interquartile range)).

## Discussion

In this study, we demonstrated that MSA-C showed various degeneration patterns from Grade 1 to 5 whereas MSA-P showed severe presynaptic degeneration patterns such as the “egg-shaped pattern” or “burst-striatum pattern.” Indeed, studies have reported that even in patients with MSA-C without parkinsonism, striatal binding in ^123^I-FP-CIT SPECT was reduced when compared with healthy controls [9]; moreover, there is no difference in response to levodopa between patients with pathologically striatonigral degeneration-type and olivopontocerebellar atrophy-type MSA [10]. In addition, we demonstrated that patients with MSA and LID tended to show severely decreased SBRs on ^123^I-FP-CIT SPECT. Interestingly, all patients with MSA (regardless of MSA-P or MSA-C) developed LID showed the “burst-striatum pattern”. These findings may support the hypothesis that severe presynaptic degeneration is necessary for LID development. From a pathophysiological perspective, in PD, presynaptic dopaminergic denervation is presumed to be an important cause of LID [7,11,12]. With the progression of nigrostriatal neuron degeneration, the serotonergic terminals metabolize levodopa to dopamine; however, these cannot store dopamine, and in turn result in the pulsatile release of dopamine based on changes in dopamine blood concentration [13]. Therefore, the same scenario may be possible in MSA. In addition to the severe loss of dopaminergic neurons, postsynaptic cell survival in the putamen may be related to both levodopa responsiveness and LID development. Although MSA usually affects postsynaptic striatal neurons as well as presynaptic dopaminergic terminals, and levodopa responsiveness is much lower than in PD, a clinicopathological study has reported that 15–20% of patients with MSA have good levodopa responsiveness, and 7–8% may develop LID [14]. In addition, putaminal damage has been reported to be less severe in MSA with levodopa responsiveness [10], and levodopa responsiveness in MSA is inversely related only to putaminal damage but not to nigral damage [14]. Indeed, our data shows higher LED associated with LID, regardless of MSA subtype.

Additionally, there may be some change in postsynaptic striatal neurons because postsynaptic hypersensitivity plays an important role in LID development in PD [11]. Thus, it is speculated that despite early nigrostriatal denervation, the preservation and hyperactivation of postsynaptic striatal neurons may be related to LID development in MSA.

Another possible hypothesis is that a change in DAT-SPECT may be related to confounding factors such as clinical features. Patients with MSA and LID had significantly longer disease durations and higher LED and levodopa responsiveness than patients with MSA without LID. Our results also revealed that LID developed earlier in MSA (5–6 years from disease onset) than in PD (about 8 to 9 years), which is in line with the findings of a previous report [3]. Visual assessment grading on DAT-SPECT correlated with parameters such as disease duration, especially for MSA-P and levodopa responsiveness. These results are also in line with those of previous studies that showed younger onset age, higher levodopa dose, and lower DAT activity to be predictors of LID development in PD [7]. Nevertheless, clinical features may also be related to the pathological changes.

Our study had several notable limitations. This was a single center retrospective open label study which featured a small sample size. Additionally, there was no quantitative analysis of the images; this should be considered as a methodological addition to future studies. Second, the diagnosis of probable or possible MSA was based on clinical information, and lacked pathological confirmation. In addition, the variability of MSA subtype prevalence statistics likely depended on the center, and this should be considered. Finally, we did not evaluate other clinical symptoms—such as detailed dysautonomia—unrelated to diagnosis and the accurate assessment of ataxia according to the International Cooperative Ataxia Scale, Scale for the Assessment and Rating of Ataxia, or the Unified Multiple System Atrophy Rating Scale [15,16,17]. Further prospective studies utilizing presynaptic and postsynaptic imaging and prospective follow-up with autopsy confirmation will be needed to confirm our findings.

## Conclusion

Our results revealed that MSA with LID severely affected presynaptic dopaminergic neurons, as seen on DAT-SPECT imaging. Higher doses of dopaminergic medication were associated with LID in MSA. This may suggest that the preservation of postsynaptic neurons may play an important role in LID development, in addition to presynaptic dopaminergic degeneration. Furthermore, slower progression with longer disease duration might be associated with LID development in MSA as well as PD. However, these hypotheses should be confirmed in future studies.
